# In Situ Visualization for 3D Ultrasound-Guided Interventions with Augmented Reality Headset

**DOI:** 10.3390/bioengineering8100131

**Published:** 2021-09-25

**Authors:** Nadia Cattari, Sara Condino, Fabrizio Cutolo, Mauro Ferrari, Vincenzo Ferrari

**Affiliations:** 1 Department of Translational Research and of New Surgical and Medical Technologies, University of Pisa, 56126 Pisa, Italy; mauro.ferrari@med.unipi.it; 2 EndoCAS Centre for Computer-Assisted Surgery, 56124 Pisa, Italy; fabrizio.cutolo@unipi.it (F.C.); vincenzo.ferrari@unipi.it (V.F.); 3 Dipartimento di Ingegneria dell’Informazione, University of Pisa, 56122 Pisa, Italy

**Keywords:** augmented reality, head-mounted display, 3D ultrasound, high-precision manual task, in-depth guidance

## Abstract

Augmented Reality (AR) headsets have become the most ergonomic and efficient visualization devices to support complex manual tasks performed under direct vision. Their ability to provide hands-free interaction with the augmented scene makes them perfect for manual procedures such as surgery. This study demonstrates the reliability of an AR head-mounted display (HMD), conceived for surgical guidance, in navigating in-depth high-precision manual tasks guided by a 3D ultrasound imaging system. The integration between the AR visualization system and the ultrasound imaging system provides the surgeon with real-time intra-operative information on unexposed soft tissues that are spatially registered with the surrounding anatomic structures. The efficacy of the AR guiding system was quantitatively assessed with an in vitro study simulating a biopsy intervention aimed at determining the level of accuracy achievable. In the experiments, 10 subjects were asked to perform the biopsy on four spherical lesions of decreasing sizes (10, 7, 5, and 3 mm). The experimental results showed that 80% of the subjects were able to successfully perform the biopsy on the 5 mm lesion, with a 2.5 mm system accuracy. The results confirmed that the proposed integrated system can be used for navigation during in-depth high-precision manual tasks.

## 1. Introduction

Augmented Reality (AR) gives the user the sense that virtual objects co-exist with real ones in the physical world. In fact, the user’s perception of the surrounding environment is enhanced by overlaying contextually relevant computer-generated information, thus providing an interactive experience [1,2]. AR is thus a key asset for developing new human–computer interaction paradigms, especially for assistance/guidance in high-precision manual tasks.

AR systems based on head-mounted displays (HMDs) provide the user with an egocentric perception of the augmented workspace and allow a hands-free interaction with it. They are thus considered to be the most ergonomic and efficient visualization devices for supporting complex manual tasks performed under direct vision, e.g., in surgery [3,4]. In fact, AR HMDs provide the physician with a surgical scene enriched with computer-generated elements derived from the imaging dataset, which is contextually merged with the real surgical scenario (i.e., in situ visualization) [5,6].

There are two types of AR: video see-through (VST) and optical see-through (OST) [7]. In VST displays, the user’s direct view is blocked, as it is mediated by one or two camera(s) rigidly anchored to the device. On the other hand, the view provided by the OST remains almost unaltered. Therefore, in OST devices, it is sufficient to render only the virtual content. By contrast, VST devices require further processing of the images acquired by the camera(s), which have to be blended with the virtual information before being projected onto the displays. Today, one of the principal research goals of computer-assisted surgery is to introduce such devices into the routine surgical workflow. However, various technological and human-factor limitations still hinder the standard adoption of these systems as surgical tools. The following provides a list of the most relevant ones, highlighting the differences between VSTs and OSTs:

- OST devices provide a full-scale resolution of the real scenario, which, in VST devices, depends on the resolution of the mediating camera.
- OST visors present an intrinsic virtual-to-real latency due to the time needed to render the virtual elements, which leads to them being spatially and temporally misaligned. On the other hand, VSTs enable the real scene to be delayed in order to match the virtual content, thus ensuring both accurate spatial and temporal virtual-to-real registration, at the expense of a higher system lag.
- In OST devices, the perceptual conflict due to the interaction between real and virtual elements [8,9,10] projected at a fixed distance (generally between 2 m and infinity) fails to stimulate natural eye accommodation. On the other hand, in VSTs, reality and virtual contents are projected at the same focal length.
- The sub-optimal ergonomics (e.g., bulkiness and weight) of both OSTs and VSTs prevents long-term use.
- OST visors need an accurate user-specific calibration mechanism between the virtual camera and the user’s eye in order to ensure the correct virtual-to-real alignment; whereas, for VST visors, the calibration procedure does not need to be user-specific, as it solely concerns the real and virtual camera [11].
- The lack of a standardized software framework for surgical application [12,13].

OST devices with a single focal plane fail to stimulate natural eye accommodation. This leads to visual fatigue and reduced user performance in completing a task that requires simultaneous focusing of real and virtual information [14,15]. Since, in this work, we aim to test the effectiveness of AR systems in guiding high-precision manual tasks, we decided that it would be more appropriate for our purposes to use a VST visor, thus ensuring a higher level of accuracy and finer usability. We used an HMD recently developed within the European project VOSTARS [16] (video and optical see-through augmented reality surgical systems, project ID: 731974). The aim of the project was to design and develop a new-concept hybrid headset, along with a software framework, for AR-based surgical navigation. The headset and the surgical navigation platform are detailed in [13] and both have already been used to guide complex shallow 3D trajectory tracing tasks on a 3D-printed replica of bony anatomies [17] and on real patients to aid the surgeon while performing Le Fort 1 osteotomies in craniomaxillofacial surgery [18]. The positive results supported the claim that the AR headset could be used for surgical navigation in guiding shallow high-precision manual tasks.

In this work, we assessed the efficacy of wearable VST AR devices for guiding in-depth high-precision manual tasks, such as needle localization of occult lesions before surgical biopsy, biopsy interventions, or cyst drainage. Learning and performing these interventions under ultrasound guidance is challenging. The ultrasound data are typically displayed on a conventional video monitor, which forces the clinician to shift his/her gaze away from patients and therefore requires considerable hand–eye coordination.

Of the ultrasound probes currently available, 2D probes are mostly used for these types of intervention. These probes provide a real-time, single-slice ultrasound image, which is displayed on the remote 2D monitor. This means that the surgeons have to have a good sense of the three-dimensional relationship, as they have to mentally superimpose the ultrasound slice over the patient to determine both the position and orientation of the needle with respect to the slice. To obtain a good three-dimensional vision of the anatomical area of interest, one possibility is to scan the area continuously and over several planes. However, keeping both the needle and the lesion within the same ultrasound slice is problematic.

Overcoming such issues entails using AR HMDs to simplify the learning and performing of ultrasound-guided interventions [19,20,21,22]. Head-mounted displays (VST in [19,20,21] and OST in [22]) are used to visualize data from a 2D ultrasound probe. The ultrasound image is directly superimposed on the patient’s anatomy in a 1:1 ratio so that the hand–eye coordination process is simplified as “the operator can directly aim the tip of the needle into the ultrasound image” [22]. Rosenthal et al. [21] made a quantitative study of 50 biopsies (25 performed with the traditional approach and 25 with the aid of an AR VST headset) and showed that the mean error in terms of deviation from the desired target was statistically significantly smaller in the HMD method than in the standard one. However, a common limitation of all the aforementioned studies is that, to guide the needle into the anatomy, the user has to adjust the position and orientation of the probe to keep the needle tip visible in the 2D ultrasound image, which requires manual dexterity and spatial coordination.

In this study, we propose the integration of a commonly available 3D ultrasound imaging system with a wearable VST AR visor. In real-time, the system shows the 3D model of the target (extracted from the 3D ultrasound volume) superimposed on the patient’s anatomy. It also shows two crosshairs that guide the user in the puncture and insertion of the needle according to the planned entry point and insertion trajectory. The aim is to eliminate any hand–eye coordination problems and, at the same time, to improve the three-dimensional perception of the patient’s anatomy, thus improving targeting accuracy.

To evaluate the feasibility of our idea, we studied and implemented custom-made calibration and tracking methods and verified that the global AR registration error is consistent with the clinical needs. To assess the efficacy of our solution, we designed an experimental study that simulates an ultrasound-guided biopsy task. We performed the biopsy task on a tailor-made phantom, using the AR surgical navigation platform provided by the VOSTARS project for the in situ visualization of medical imaging data derived from a Philips 3D ultrasound acquisition system.

## 2. Materials and Methods

In this section, we detail the hardware and software components for the integration of the 3D ultrasound acquisition system and the AR platform. We also describe the in vitro testing performed to evaluate the accuracy and precision of our system.

### 2.1. Ultrasound Acquisition System

Similarly to magnetic resonance imaging (MRI), 3D ultrasound provides volumetric information on the patient’s anatomy, preventing exposure to the procedural radiation dose for both patients and clinical staff. However, in contrast to MRI, 3D ultrasound does not require a dedicated room; it provides immediate results; it is less expensive; and it is portable. This means that it can be used in both extra-operative (e.g., surgical training) and intra-operative environments.

**Figure 1:**
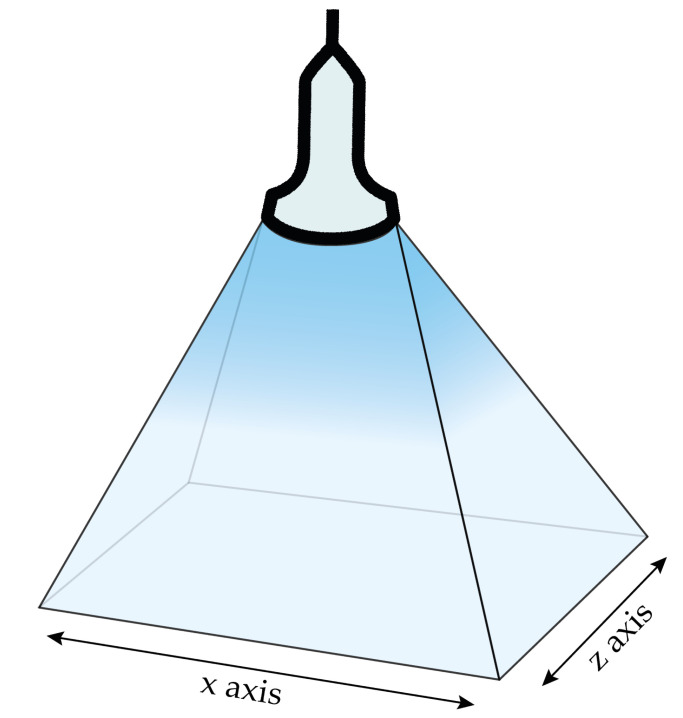
Acquisition volume of the VL 13-5 ultrasound transducer with the associated Cartesian axes.

We used a 3D ultrasound transducer (VL 13-5) and a Philips iU22 ultrasonic system [23] (Philips Medical Systems, N.A.; Bothell, WA, USA) to generate DICOM files that can be easily extracted and read by image processing software. The dimension of the volumes acquired in our experiments was approximately 38 mm × 91 mm × 27 mm along the x-axis, y-axis, and z-axis, respectively. Figure 1 depicts the Cartesian axes associated to the ultrasound transducer acquisition volume.

### 2.2. Hardware and Software Components of the AR Platform

This subsection describes the hardware and software components of the HMD-based AR surgical navigation platform developed within the VOSTARS project.

#### 2.2.1. Head-Mounted Display

The aim of the VOSTARS project was to develop a new AR headset that could exploit both OST and VST mechanisms. The headset also had to meet rigorous technological requirements in order to mitigate the perceptual conflicts that typically occur with commercially available AR headsets during close-up activities. This goal was achieved by re-engineering a commercial binocular OST visor (ARS.30 by Trivisio [24]) with a similar approach to that described in our previous work [25].

The switching between the two see-through mechanisms is provided through a pair of liquid-crystal (LC) optical shutters placed in front of the displays, which can be electronically dimmed to change their transparency. The VST camera-mediated view is provided by a pair of world-facing RGB cameras rigidly incorporated within the HMD. The cameras have an anthropometric interaxial distance (∼6.3 cm) and a fixed convergence angle of 3.4${}^{\circ }$. This provides sufficient stereo overlap at about 40 cm (i.e., an average working distance for manual tasks) and mitigates the horizontal disparity due to camera-to-eye parallax. The stereo camera pair is composed of two synchronized LI-OV4689 cameras (Leopard Imaging Inc., Fremont, CA, USA), equipped with a 1/3^″^ OmniVision CMOS 4 M pixel sensor (OmniVision, Santa Clara, CA, USA) and M12 lens support (Edmund Optics, Barrington, NJ, USA) with a focal length of 6 mm, which was chosen to restore the 1:1 scale factor at arm’s distance.

**Figure 2:**
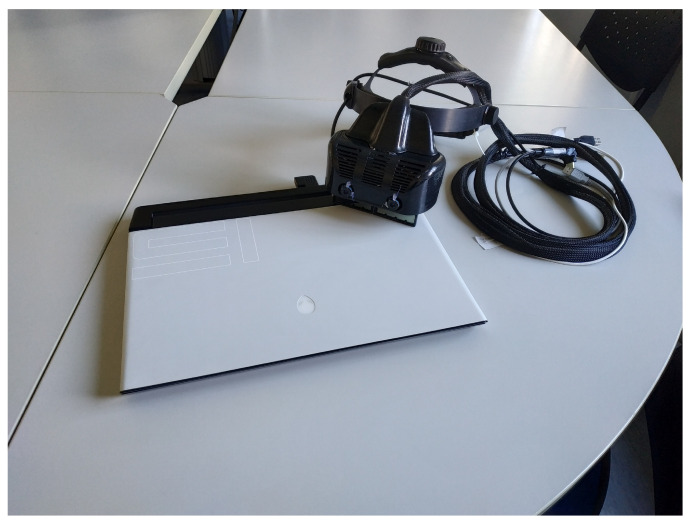
The custom-made hybrid optical/video see-through head-mounted display and the laptop.

The computing unit runs on a laptop with an Intel Core i7-8750H CPU@2.20 GHz with 12 cores and 16 GB RAM (Intel Corp., Santa Clara, CA, USA) and an Nvidia GeForce RTX 2060 (6 GB) with 1920 CUDA Cores (Nvidia Corp., Santa Clara, CA, USA) graphic card processing unit. Figure 2 shows the laptop and the VOSTARS headset used.

#### 2.2.2. AR Software Framework

The software framework is designed to support in situ visualization of medical imaging data using the VTK library, an open-source library for 3D computer graphics, modeling, and volume rendering. The software features an inside-out optical tracking algorithm based on OpenCV API 3.4.1, which performs the stereo localization of a triple of spherical markers, using our ad hoc algorithm [13]. The tracking algorithm has an excellent frame rate and tracking accuracy, as already proved in our previous works [13,17]. The software exploits tracking information to augment the scene, registers the virtual content onto the reality, and renders the augmented frames onto the displays. The pose of the virtual object is constrained to the pose of the tracked triple, thus ensuring locational realism. In addition, specifically for VST modality, the software processes the images of the real scene captured by the cameras and merges these images with the virtual content before rendering. To ensure the AR registration, the linear projection parameters of the virtual rendering cameras are set to the intrinsics of the real camera. The non-linear distortion introduced by the optics of the real camera is compensated for by undistorting the video frames using the camera intrinsics.

To summarize, the software provides the locationally registered in situ visualization of task-oriented digital content in terms of both optical and video see-through-based augmentations. The framework is highly configurable in terms of tracking capabilities and renderable content and ensures significant computational efficiency, thanks to the CUDA-based multi-threaded architecture. This architecture enables an average frame rate of ∼30 fps to be obtained for each eye.

### 2.3. 3D Ultrasound System Integration with AR Platform

**Figure 3:**
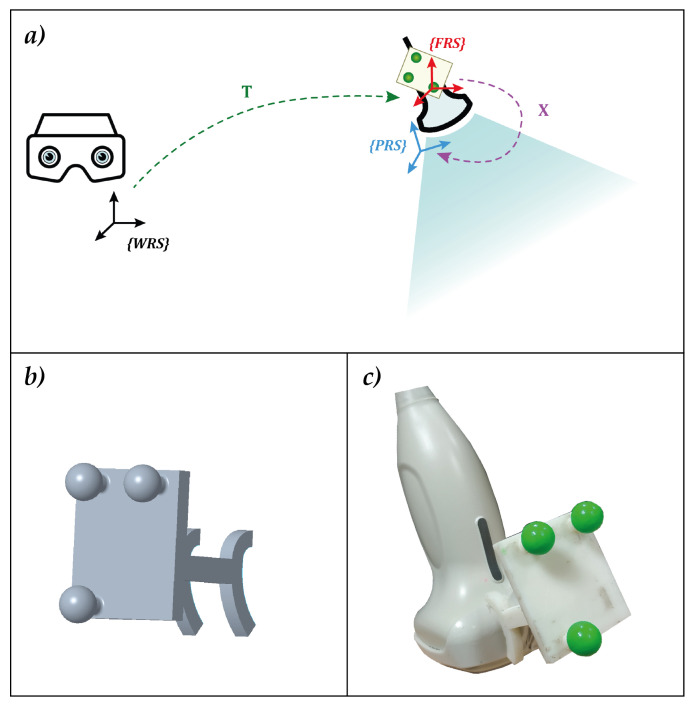
(**a**) the elements involved in the calibration with the associated reference systems and their relative transformation matrices. (**b**) the optical frame designed for calibration. The three spherical markers were dyed in fluorescent green to improve the robustness of the RGB tracking (**c**).

The integration between the 3D ultrasound acquisition system and the AR platform required the development of a MATLAB (R2018b MathWorks, Inc., Natick, MA, USA) dedicated calibration routine. For the probe to be used in combination with the AR platform, and thus, for the ultrasound virtual content to be correctly spatially registered over the real scene, the relative pose between the AR visor and the acquisition volume of the ultrasound probe needs to be known at all times. This entails:

- The definition of a local reference system associated with the ultrasound probe (FRS in Figure 3a).
- The tracking of the ultrasound probe pose in the world reference system associated with the visor (WRS in Figure 3a), i.e., the definition of the transformation matrix between WRS and FRS (transformation *T* in Figure 3a).
- The definition of the transformation matrix *X* between FRS and the reference system of the ultrasound acquisition volume (PRS in Figure 3a).

Figure 3a shows all the elements needed and their relations. To meet the first two requirements, an optical frame, shown in Figure 3b,c, was designed, printed, and uniquely anchored to the probe. The optical tracking algorithm provided by the AR software framework introduced in Section 2.2.2 was exploited to track the position of the probe in real-time.

With regard to the third requirement (i.e., the definition of the transformation matrix), we had to identify the coordinates of the salient points acquired within the ultrasound volume with respect to the reference system of the probe itself. This is usually performed by imaging a phantom, namely an object with known physical properties and dimensions. Following [26], calibration methods can be classified according to the phantom type used. The basic concept is to exploit the invariant point, line, or plane by using one or multiple small metal spheres [27], crossing wires [28,29,30], or using the supporting structure of the phantom itself [30]. Since there is still no agreement as to which phantom design is the best, we created a customized calibration phantom.

**Figure 4:**
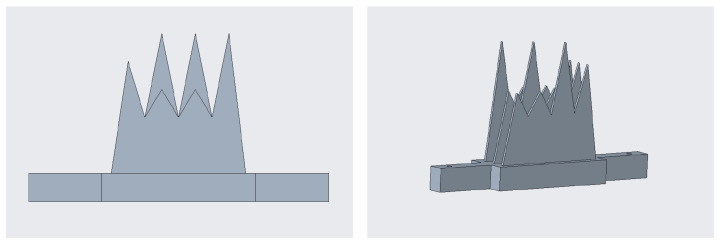
The ad hoc phantom designed for the calibration: frontal view on the (**left**), and perspective view on the (**right**).

Figure 4 shows the phantom, hereafter referred to as the comb, owing to its shape. It was designed with computer-aided design (CAD) software (Creo Parametrics 6.0) and manufactured in acrylonitrile butadiene styrene (ABS) using a 3D printer (Statasys, Eden Prairie, MN, USA). A MATLAB subroutine was created to register the 12-tipped comb vertices, used as fiducial or salient points, using a singular value decomposition (SVD) algorithm [31]. The comb was dipped in a water bath at 37° (i.e., the average body temperature, to simulate the real conditions of an ultrasound) and acquired with the ultrasound volume from multiple steady probe positions to fulfill this calibration step. The twelve tips of the comb were extracted from each acquisition with dedicated segmentation software (ITK-SNAP), and the registration between these point clouds and the coordinates of the same points derived from the CAD file was performed.

Finally, we defined the transformation matrix that links the reference system of the probe (PRS) to the reference system of the optical frame (FRS). For this purpose, an additional MATLAB subroutine was created, which incorporates the hand–eye calibration method by Park [32].

**Figure 5:**
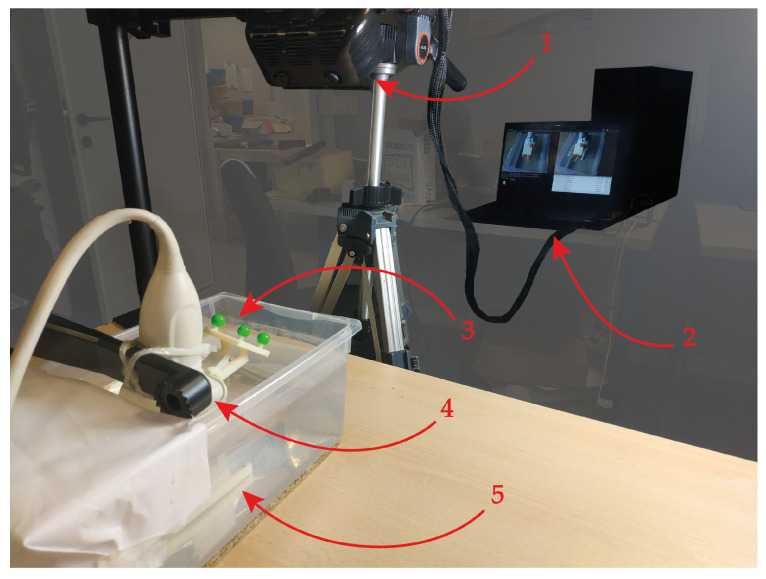
Set-up implemented for the calibration: 1 → The augmented reality head-mounted display employed. 2 → The laptop running the RGB tracking algorithm. 3 → The optical frame anchored to the ultrasound probe. 4 → The mounting arm holding the ultrasound probe, which enables it to be moved in space. 5 → The calibration comb dipped in the 37° water bath.

Figure 5 shows the setup for the calibration. The AR HMD is fixed by a tripod, while the comb used in the previous step is dipped in a water bath at 37° and set within the field of view of the visor. The ultrasound probe was attached to a mounting arm in order to capture the comb from different perspectives within the field of view of the visor. This calibration yielded a homogeneous matrix equation of the form:

(1) AX=XB

in which:

- *X* is the unknown transformation between the optical frame and the ultrasound probe.
- *A* is the homogeneous transformation that describes the relative pose between two consecutive poses, henceforth referred as T, of the optical frame anchored to the ultrasound probe, in the world reference system (WRS) associated with the HMD.
- *B* is the homogeneous transformation that describes the relative pose, henceforth referred as U, between two consecutive poses of the comb (CRS) in the PRS.

**Figure 6:**
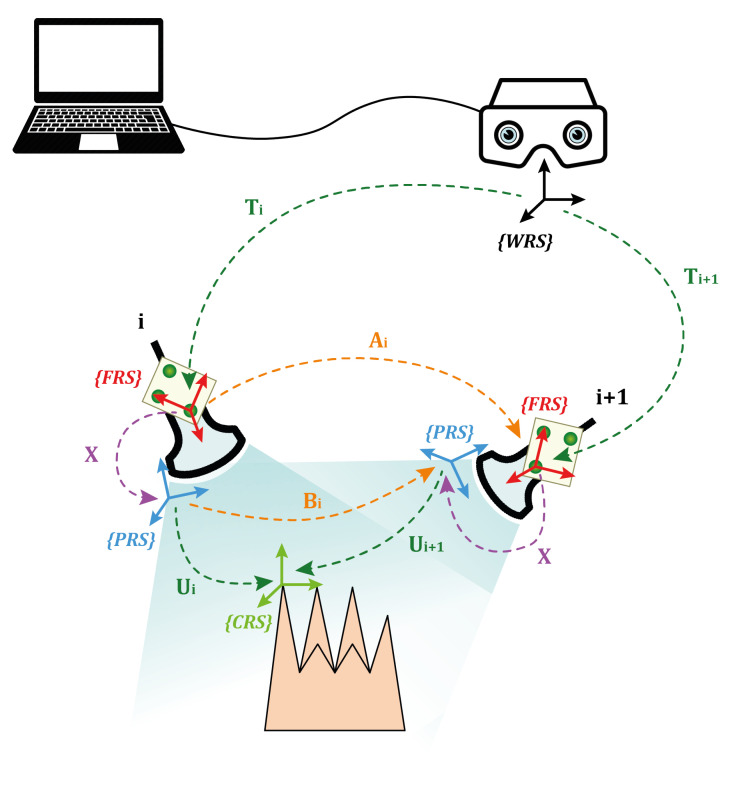
Illustration of the *AX* = *XB* procedure for the probe-to-optical frame calibration.

The ultrasound probe was moved in 15 different positions; therefore, transformation matrices *A* and *B* were stored with the 14 relative poses, derived from two consecutive time instants, pose at time *i*+1 and pose at time *i*. In Figure 6, $A_{i}$ denotes the motion between $T_{i}$ and $T_{i+1}$, whereas $B_{i}$ denotes the motion between $U_{i}$ and $U_{i+1}$. Hence, Equation (1) can be rewritten as:

(2) $$A_{i}X=XB_{i};i=1,\ldots ,15$$

where $A_{i}$ and $B_{i}$ can be calculated as:

(3) $$A_{i}=\left({\left(T_{i}\right)}^{-1}\times T_{i+1}\right)$$

(4) $$B_{i}=\left(U_{i}\times {\left(U_{i+1}\right)}^{-1}\right)$$

Once *X* is derived, the calibration procedure is finished.

### 2.4. System Evaluation

The evaluation of the system included a quantitative analysis of the accuracy of the AR overlay and a user study to estimate the accuracy achievable during an in-depth high-precision task. The following subsections describe the experimental setups and the protocols implemented to test the system.

#### 2.4.1. Echogenic Materials

System testing entailed selecting an echogenic material with acoustic properties resembling those of human tissue. Particular attention was paid to the speed of sound in the material, which is crucial to preventing distortions of shape and size during reconstruction, and to the stability of the acoustic properties over time.

There are various material for creating ultrasound phantoms, from the most common organic materials such as agar, agarose, gelatine, gellan gum, and polyvinyl alcohol (PVA), to non-organic materials, such as polyvinyl chloride plastisol (PVC-P), room-temperature-vulcanizing (RTV) silicones, polydimethylsiloxane (PDMS), and polyurethane (PU). Biopolymers contain a high percentage of water (>80%), which makes them similar to soft biological tissues [33]. However, they are prone to water evaporation and bacterial growth and are therefore not suitable for long-term use and storage [34]. On the other hand, chemically synthesized polymers are more stable and durable [35,36,37]; although, the lack of water makes them less similar to real tissues.

Polyvinyl chloride plastisol is the best candidate for ultrasound phantom manufacturing. Compared with biopolymers, PVC-P is resistant to bacterial attack, while compared with chemically synthesized polymers, it has clear acoustic advantages over silicones and PDMS for ultrasound imaging [38,39]. In addition, the speed of sound in PVC-P is 1400 m/s (very close to 1500 m/s, namely, the average speed of sound in human tissue), but it can be increased up to 1490 m/s with the addition of graphite. Finally, mixing PVC-P creates phantoms with different degrees of hardness and workability. For these reasons, PVC-P was selected as the echogenic material for manufacturing ultrasound phantoms in this study.

#### 2.4.2. Quantitative Evaluation of AR Overlay Accuracy

The AR overlay accuracy can be assessed by calculating the 2D target visualization error (TVE2D), namely the offset, expressed in pixels, between the real and the virtual objects in the image plane, evaluated as the reprojection error in 2D onto the displayed AR image.

To quantitatively evaluate the AR overlay accuracy, tests were performed on an ad hoc developed ultrasound phantom in the shape of a spherical lollipop (20 mm in diameter). The phantom was manufactured in PVC-P, with a supporting structure made of ABS. The choice of shape is linked to the ease of identifying the centroids of spherical objects, both real and virtual, which made it easier to assess the accuracy of the AR overlay. To create the virtual sphere to be registered over the head of the lollipop, the phantom was dipped in the usual 37° water bath, and the ultrasound probe was positioned to have the head of the lollipop centered in the acquisition volume, thus mimicking the positioning of a target lesion to be biopsied in standard protocols.

**Figure 7:**
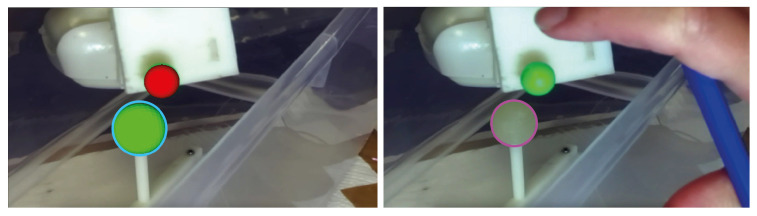
Example of image processing for detecting real and virtual spheres. On the (**left**), the AR image: the real scene is augmented with the virtual sphere (in green) registered over the lollipop’s head and the tracked optical frame (in red). The detected virtual sphere is represented by a blue circle. On the (**right**), the real image: the detected real lollipop’s head is represented by a magenta circle.

Once calibrated, the AR HMD was anchored over a flexible mounting arm and moved in 10 different positions to simulate plausible user points of view. For each pose of the visor, four different images were acquired: three AR images and a real image with the AR switched off, with a total of 40 images. This set of images was processed in Matlab to determine the centroids of both virtual and real spheres through the *imfindcircles* function, and thus to evaluate the TVE2D, namely the offset of the two centroids in the image plane. To facilitate the work of the algorithm, the real image was cropped around a region of interest containing solely the real sphere. Figure 7 shows an example of the AR and real image to detect the spheres.

The target visualization error in 3D (TVE3D), which is the estimation at a fixed distance of the visualization error in space (in terms of Euclidean distance), was derived from the TVE2D by proportioning the sphere’s size in pixels for each image with the actual size of the lollipop’s head.

#### 2.4.3. User Study—Accuracy of 3D Ultrasound-Guided Biopsy

The level of precision and accuracy that the integrated system can ensure in performing an in-depth high-precision task was also checked. Ten participants were recruited and were asked to perform a simulated 3D ultrasound-guided biopsy procedure in a non-anthropomorphic parallelepiped-shaped phantom.

**Figure 8:**
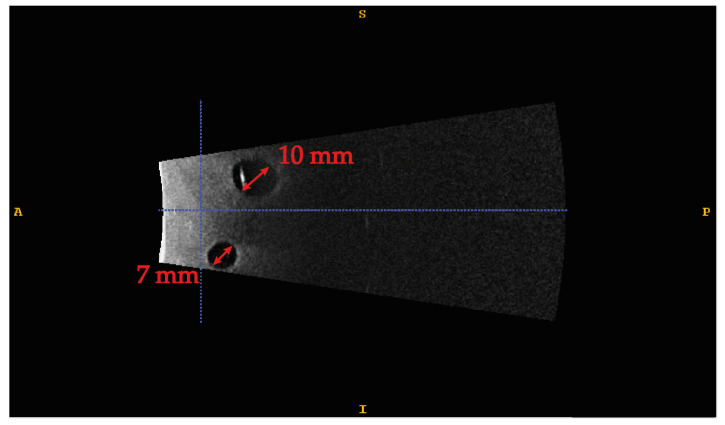
Ultrasound image of the phantom. Two anechoic lesions, 10 and 7 mm, respectively, are detected in the acquired volume.

The phantom is made of PVC-P doped with 2% by weight with graphite to increase the speed of sound in the material to make it comparable to human tissues. Four different-sized spherical lesions were randomly included to evaluate the system’s accuracy in guiding an in-depth high-precision manual task. More specifically, sizes of 3, 5, 7, and 10 mm in diameter were used to test an accuracy level of 1.5 mm, 2.5 mm, 3.5 mm, and 5 mm, respectively. Figure 8 shows the ultrasound image of two of the four lesions embodied in the phantom. The range of target lesion size was chosen according to the clinical needs and the results of the accuracy evaluation in terms of TVE3D reported in Section 3.1. More specifically, the maximum size is equal to the average size used for histopathological diagnosis biopsies [40], whereas the minimum size is compatible with the maximum accuracy, in terms of TVE3D, plus a potential inaccuracy factor introduced by users. The lesions were made of non-doped PVC-P, to make them akin to real anechoic lesions, and were used as targets in our manually performed task.

Ten participants with normal or corrected-to-normal visual acuity were recruited among university students, staff, and faculty members. None of the participants had previous experience in performing an ultrasound-guided biopsy.

**Figure 9:**
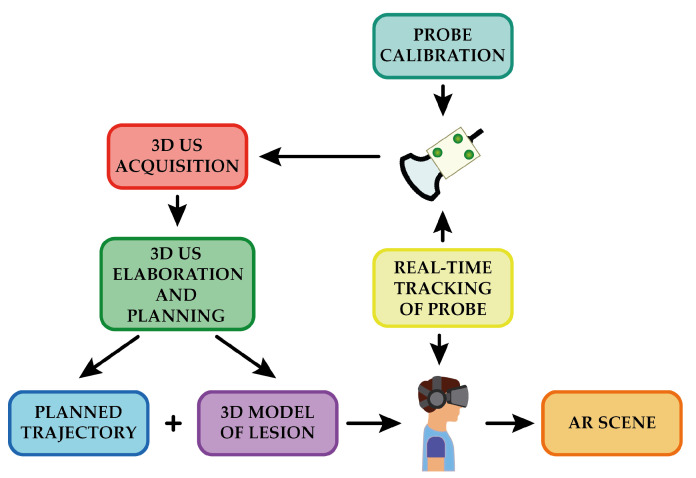
Overview of the workflow for the generation of the AR scene.

Due to the phantom material’s excellent healing capabilities, it was possible to use a single phantom containing the four lesions for all of the users. For each lesion embodied in the phantom, a volume of 38 × 91 × 27 mm^3^ was acquired, and the lesion was extracted via segmentation. The segmented mesh was then transformed into a virtual reality modeling language (VRML) file ready to be uploaded to the AR platform and displayed onto the HMD, coherently registered to the real phantom. Figure 9 shows the overall workflow for the creation of the AR scene.

Each participant was provided with a 21 G syringe (MTD Medical Technology and Devices S.A., Lugano, Switzerland) (0.8 mm external diameter) and was asked to wear the HMD and to perform the biopsy task reaching the target lesion with the tip of the needle. The participants were also asked to mention if they perceived any visual discomfort or any spatial displacement of the virtual content while performing the task.

**Figure 10:**
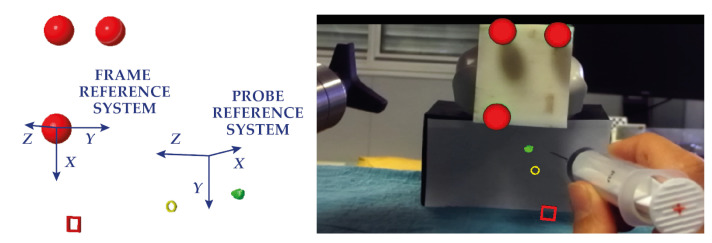
Augmentation of the real scene. On the (**left**), the 3D CAD modelling of the virtual elements used to augment the real scene. On the (**right**), the AR image visualized through the HMD.

To guide the needle insertion, the lesion concerned and two crosshairs were virtually added to the augmented scene and were thus visible through the visor. This visualization technique has already been tested in other studies and is successful in guiding high-precision tasks in different anatomical districts, such as neuro- and spine surgery [41,42]. One circular crosshair was placed on the surface of the phantom, and one square crosshair was placed 13 cm away from the surface (approximately the length of the syringe). Both crosshairs were aligned by the user along the ideal insertion trajectory. The participants had to line up their monocular viewpoint so that the circular crosshair was inscribed within square one, and both were in line with the lesion. Subsequently, they had to point the tip of the needle at the center of the two crosshairs and align the syringe along the trajectory by means of a cross drawn on the bottom of the syringe. The lesion depth was indicated with colored markings on the needle. Figure 10 shows the AR image visualized through the visor.

**Figure 11:**
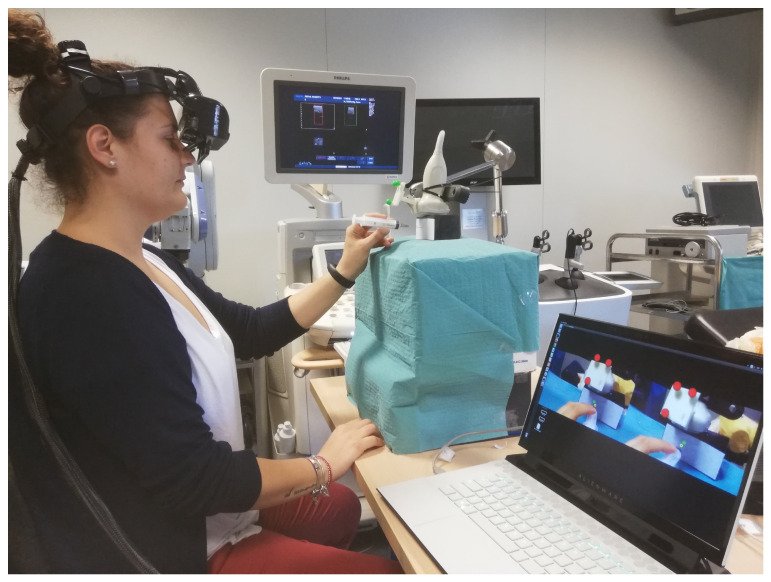
Experimental setup with a user while performing the biopsy task. In the lower right corner are the augmented images projected onto the displays, whereas, on the Philips machine monitor, the real-time ultrasound acquisition can be seen.

The participants conducted four different sessions, each session involving one of the four target lesions at a time. The objective was considered achieved if the real-time ultrasound image showed the needle within the target lesion. The participants were given only one chance to perform the biopsy on each lesion. Figure 11 illustrates a subject while performing the biopsy task.

## 3. Results

### 3.1. AR Overlay Accuracy Results

The results of the AR overlay accuracy test are reported in Table 1. The mean TVE2D obtained was 8.69 px, with a minimum value of 4.08 px and a maximum value of 10.95 px, which corresponded to a mean TVE3D of 2.02 mm, with a minimum of 0.87 mm and a maximum of 2.79 mm.

### 3.2. 3D Ultrasound-Guided Biopsy Results

All 10 participants completed the four biopsy tasks without perceiving any visual discomfort or any spatial displacement of the virtual elements. Table 2 reports the success ratio in completing the tasks for each participant and each target lesion. All of the participants were able to perform the biopsy task within an accuracy of 5 mm, 90% of them performed it within an accuracy of 3.5 mm, 80% of the subjects stayed within an accuracy of 2.5 mm, and finally, 40% of the subjects were able to achieve an accuracy of up to 1.5 mm.

## 4. Discussion

We proposed the use of an AR surgical navigation platform and a customized AR headset for the guidance of in-depth high-precision manual tasks. We addressed the navigation of 3D ultrasound image-guided interventions, such as biopsies, with the in situ visualization of the target. To this end, we developed a dedicated calibration routine, to integrate the data from the 3D ultrasound imaging system within the AR platform, and thus the AR visor.

We designed an experimental study to quantitatively evaluate the accuracy level that the integrated system could provide in guiding a simulated biopsy task. First, we analyzed the TVE to determine the intrinsic accuracy achievable in the AR visualization, obtaining a TVE3D ranging from 0.87 mm to 2.79 mm, with a mean value of 2.02 mm. To also consider any user inaccuracy, we designed a user study and engineered an ad hoc ultrasound phantom with different-sized lesions randomly enclosed within it. The results of this second test, given in terms of success rates, suggest that the integrated system could be successfully used to guide in-depth high-precision manual tasks. Despite their inexperience in performing the biopsies, all of the participants were able to reach the target lesion of 10 mm, which is the average lesion size targeted during histopathological diagnosis biopsies [40], highlighting a targeting accuracy of 5 mm. The results obtained with the remaining lesions are comparable with those obtained with robotic guidance systems. The robotic system proposed by Welleweerd et al. [43] can perform a breast biopsy task within a level of accuracy of 3.03 mm, measured in terms of the Euclidean distance between the needle and target. In our study, 90% of the subjects performed the task with an accuracy of 3.5 mm. In addition, 80% of the participants were able to stay within an accuracy of 2.5 mm, and 40% achieved an accuracy of 1.5 mm. We assume that the latter result, which was poorer than the others, could be due to errors in the integration chain (e.g., the low resolution offered by the 3D ultrasound scanner, the tracking error of the optical frame attached to the probe, and the calibration error), which together represent the lower limit of accuracy of our system.

One limitation of this work is due to a technological issue. To date, ultrasound imaging does not enable the real-time video streaming of volumetric acquisitions. These can only be stored on a disk and exported for external processing. For this reason, in the experimental setup, we were forced to constrain the probe in a predetermined pose; thus, the participants were not given the opportunity to orient it as they wished. Another limitation is the time needed to prepare the setup. Currently, the time required for preparation varies from 20 to 25 min. The goal of future studies is to reduce the waiting time (probably to the order of a few minutes) by automating the generation of the AR content, starting from segmentation. Ideally, exporting the acquired volume in real-time would positively boost the waiting time reduction.

In conclusion, the results of this study show the potential of integration of a 3D ultrasound imaging scanner and AR visualization system (software framework combined with a dedicated headset) for the guidance of in-depth high-precision manual tasks. We also provided guidelines on how to perform the calibration between these two systems and on the materials that can be used to fabricate an ultrasound phantom. We are planning to investigate whether the proposed integrated system could decrease both the inter- and intra-operator variability in performance. In addition, we will also challenge our system with the simulation of more complex in-depth tasks, such as a tumorectomy.

## Figures and Tables

**Table 1:** Results of the AR overlay accuracy test in terms of TVE2D and TVE3D.

Target Visualization Error (TVE)			
	**Mean**	**Min**	**Max**
**2D (px)**	8.69	4.08	10.95
**3D (mm)**	2.02	0.87	2.79

**Table 2:** Success rate for all the participants involved in the tests.

	Target Lesions				Success Ratio
	10 mm	7 mm	5 mm	3 mm	
**1.**	in	in	in	out	3/4
**2.**	in	in	in	in	4/4
**3.**	in	in	in	out	3/4
**4.**	in	out	in	out	2/4
**5.**	in	in	in	in	4/4
**6.**	in	in	in	out	3/4
**7.**	in	in	in	out	3/4
**8.**	in	in	out	in	3/4
**9.**	in	in	in	in	4/4
**10.**	in	in	out	out	2/4
**Success ratio**	10/10	9/10	8/10	4/10	

## Data Availability

The data presented in this study are available upon request from the corresponding author.

## Abbreviations

The following abbreviations are used in this manuscript:

ABS	Acrylonitrile Butadiene Styrene
AR	Augmented Reality
CAD	Computer-Aided Design
CRS	Comb Reference System
FRS	Frame Reference System
HMD	Head-Mounted Display
LC	Liquid Crystal
OST	Optical See-Through
PDMS	Polydimethylsiloxane
PRS	Probe Reference System
PVA	Polyvinil Alcohol
PVC-P	Polyvinyl Chloride Plastisol
PU	Polyurethane
RTV	Room-Temperature-Vulcanizing Silicone
SVD	Singular Value Decomposition
TVE	Target Visualization Error
VMRL	Virtual Reality Modeling Language
VST	Video See-Through
WRS	World Reference System
